# Baseline estrogenic activity in juvenile brown trout liver and primary hepatocytes: integrated molecular and immunohistochemical analysis

**DOI:** 10.1007/s00360-026-01657-0

**Published:** 2026-04-01

**Authors:** Inês C. S. Barbosa, Fernanda Malhão, Eduardo Rocha, Tânia Vieira Madureira

**Affiliations:** 1 Superior School of Health (E2S), Polytechnic Institute of Porto (P.PORTO), Rua Dr. António Bernardino de Almeida 400, 4200-072 Porto, Portugal; 2 Group of Animal Morphology and Toxicology, Interdisciplinary Centre of Marine and Environmental Research (CIIMAR/CIMAR), University of Porto, Terminal de Cruzeiros do Porto de Leixões, Av. General Norton de Matos S/N, 4450-208 Matosinhos, Portugal; 3 Laboratory of Histology and Embryology, Department of Microscopy, School of Medicine and Biomedical Sciences (ICBAS), University of Porto, Rua Jorge Viterbo Ferreira 228, 4050-313 Porto, Portugal

**Keywords:** Brown trout, Hepatocytes, Estrogen receptors, Vitellogenin, Zona pellucida proteins

## Abstract

Characterization of sex and biometry of fish donors for primary cell culture is often overlooked. Consequently, the influence of donor biological variability—such as reproductive status—on basal estrogenic expression in fish hepatocytes remains underexplored in toxicological studies. This study examines whether inherent differences among 1-year-old juvenile brown trout (*Salmo trutta*) are reflected in estrogenic signatures in liver tissue and the corresponding primary hepatocyte cultures from the same individuals. To our knowledge, no previous research has systematically assessed interindividual variation in estrogenic markers in brown trout primary hepatocytes. To address this, we evaluated the gene and protein expression of four classical estrogenic targets: vitellogenin, zona pellucida protein, estrogen receptor alpha, and estrogen receptor beta. No statistically significant differences in basal expression levels were observed across male and female donors in either hepatocytes or liver tissue. Nonetheless, interindividual variability was evident, reflecting natural biological differences in gonadal development and physiological status. The findings highlight the need to document and control donor sex, reproductive status, and individual variability when selecting fish for hepatocyte culture, even when sex differences in basal expression are not statistically significant, thereby providing a crucial baseline for toxicological experiments using primary trout hepatocytes.

**Supplementary Information:**

The online version contains supplementary material available at 10.1007/s00360-026-01657-0.

## Introduction

Fish in vivo studies are widely used in ecotoxicology to evaluate the effects of estrogenic compounds—either individually or in mixtures—on aquatic biota under laboratory simulation experiments (Brian et al. 2007; Orn et al. 2006; Van Belt et al. 2001; Zhang et al. 2010), and through field monitoring campaigns to locate hotspot-contaminated areas (Hecker et al. 2002; Müller et al. 2020). The growing ethical and regulatory pressure to reduce animal use has driven interest toward alternative models. In this context, in vitro fish models have gained increasing attention as promising tools for toxicological assessment (Gómez-Mercader et al. 2024; Nguyen et al. 2024).

The liver is a pivotal multifunctional organ with relevant detoxification and metabolic functions. Given its importance, fish primary hepatocytes are extensively applied to screen for the toxic impacts and the underlying mechanisms caused by distinct chemicals (Hultman et al. 2015a; Zablocki da Luz et al. 2024; Madureira et al. 2015). Importantly, the liver exhibits strong sexual dimorphism across species, reflecting different metabolic requirements for male and female reproduction in both oviparous and viviparous species (Roy and Chatterjee 1983). In oviparous species, such as fish, the female’s liver is primarily responsible for producing yolk protein precursors, e.g., vitellogenin (Vtg) and specific zona pellucida proteins (ZP) (Arukwe and Goksøyr 2003). Recent omics-based studies have confirmed sex-specific hepatic profiles in distinct fish species, including zebrafish (*Danio rerio*) (Zheng et al. 2013), Japanese medaka (*Oryzias latipes*) (Qiao et al. 2016) and spotted scat (*Scatophagus argus*) (Chen et al. 2021). Interestingly, different liver regeneration profiles have been observed between male and female zebrafish, with estrogen receptors playing a role in this disparity (Zhu et al. 2024).

Sex-specific responses are not limited to in vivo studies. In vitro assays using fish hepatocyte cultures have also reported sex-related differences, especially regarding xenobiotic metabolization and sensitivity to environmental contaminants (Winzer et al. 2002a, 2001). For example, Winzer et al. (2002a; b) studied immature European flounder (*Platichthys flesus*) of both sexes and found that female-derived hepatocytes showed a significant reduction in glucose-6-phosphate dehydrogenase (G6PDH) activity, an indicator of oxidative stress response, suggesting they were more prone to toxic injury (Winzer et al. 2002b). Further, primary hepatocytes isolated from sexually mature male and female tilapia (*Oreochromis mossambicus*) (Riley et al. 2004) and common carp (*Cyprinus carpio*) (Bickley et al. 2009) showed distinctive Vtg induction profiles under estrogenic stimuli.

Even though biological differences among donor fish can influence hepatocyte responses, they are underreported, potentially affecting the interpretation and reproducibility of xenobiotic exposure studies. Some studies using fish primary hepatocytes did not disclose donor characteristics (e.g., Zablocki da Luz et al. 2024; Zhou et al. 2006), while others focus on specific developmental or reproductive stages (Riley et al. 2004; Segner et al. 1994), immature (Hultman et al. 2015a; Winzer et al. 2001; Lopes et al. 2020), or even sterile fish (Pelissero et al. 1993). Immature fish are often used under the premise that the animal’s sex is irrelevant.

Considering the above context, the main aim of this research is to investigate whether donor-specific factors influence basal estrogenicity in the liver and primary hepatocytes of 1-year-old juvenile brown trout. To our knowledge, no prior study has systematically evaluated individual-level variation in estrogenic markers in brown trout hepatocyte cultures. To achieve this purpose, liver and primary hepatocyte cultures from the same fish donor were used to assess the gene and protein expression levels of four classic estrogenic targets. The mRNA levels of *vitellogenin A* (*VtgA*), *zona pellucida protein 2.5* (*ZP2.5*), *estrogen receptor alpha* (*ERα*), and *estrogen receptor beta* (*ERβ*) were analyzed by quantitative real-time polymerase chain reaction (RT-qPCR). Protein phenotypic anchoring was made by immunohistochemistry targeting Vtg, ZP, ERα, and ERβ. Liver samples (from the same animals) were used as an in vivo reference. This integrated approach sets fundamental baselines to support the design of in vitro toxicological assays using fish hepatocytes and underscores the relevance of accounting for biological variability—such as reproductive status—when selecting juvenile fish donors for primary cultures.

## Materials and methods

### Fish

Ten brown trout (1-year-old) from the same stock batch were purchased from a national aquaculture facility (Torno, Amarante, Portugal) in June 2024. It was ensured that fish were fasting on the day prior to the hepatocyte isolations. Fish handling and humane killing were performed following the Portuguese Decree-Laws No. 113/2013 and No. 1/2019 and the guidelines of the 2010/63/EU European Directive, and were supervised by experts accredited by the Portuguese Directorate-General for Food and Veterinary in Laboratory Animal Science according to FELASA category C recommendations.

### Primary hepatocyte isolation and sampling procedures

Each fish was euthanized with an overdose (0.6 mL/L) of an aqueous solution of ethylene glycol monophenyl ether (Merck KGaA, Darmstadt, Germany). During sampling, fish were thoroughly assessed for indications of illness, parasitic infections, or any visible physical deformities. No visible signs of health issues were detected. Fish were weighed and measured (standard and total lengths) to calculate the condition factor (K), according to: K = 100 × fish wet weight (g) / (total length)^3^ (cm) (Ricker 1975). The liver and gonads were also weighed to calculate organ-somatic indexes, as follows: organ weight (g) / fish weight (g) × 100. To avoid contamination of the cell culture with blood cells, complete blood withdrawal was performed via the caudal vein using an insulin syringe. The liver was removed and weighed, and two fragments, each weighing about 20 mg, were sampled from a peripheral zone of the organ to minimize blood vessel collapse and any potential effects on the subsequent perfusion. One liver slice was immediately immersed in 10% neutral buffered formalin (Epredia, Breda, The Netherlands) and used for histological and immunohistochemical analyses. The other fragment was snap-frozen in liquid nitrogen and stored at − 80 °C for molecular studies. The fish gonads were collected and immersed in 10% neutral buffered formalin (Epredia, Breda, The Netherlands) to confirm the sex of the fish by light microscopy. The primary hepatocyte isolation was performed using a two-step collagenase perfusion technique, optimized for brown trout and previously described in detail (Madureira et al. 2015). The cellular suspension was initially filtered through two nylon membranes of different mesh sizes (250 µm and 50 µm). After three buffer washes with consecutive centrifugation steps at 160 g for 5 min at 4 °C, the final pellet was obtained. The pellet was then resuspended in phenol red-free Leibovitz’s L15 medium (Invitrogen™, California, CA, USA), with 5% of charcoal-stripped fetal bovine serum (FBS) (Merck KGaA, Darmstadt, Germany), 100 µg/mL of streptomycin (Merck KGaA, Darmstadt, Germany) and 100 U/mL of penicillin (Merck KGaA, Darmstadt, Germany) (Madureira et al. 2015, 2016). The viability was measured using a Countess™ Automated Cell Counter (Invitrogen™, California, CA, USA), using a 1:1 dilution of cell suspension and trypan blue (Invitrogen™, California, CA, USA). The purity of the isolated hepatocytes was approximately 96%, as confirmed by transmission electron microscopy based on ultrastructural features; the remaining cells were identified as cholangiocytes.

### Primary hepatocyte culture

Primary hepatocytes from each fish were cultured at 1 × 10^6^ cells per well in 500 µL of Leibovitz’s L15 medium in 24-well plates (Orange Scientific, Braine-l’Alleud, Belgium), coated with 0.3 mg/mL of poly-l-lysine hydrobromide (Merck KGaA, Darmstadt, Germany), at 19 °C (without extra O_2_/CO_2_). Hepatocytes were cultured for 96 h in one 24-well plate, and the medium was changed every 24 h, as established in previous studies (Lopes et al. 2020). After 96 h in culture, hepatocytes were trypsinized using trypsin/EDTA (Biowest, Nuaillé, France) and cell viability was checked with a Countess™ Automated Cell Counter (Invitrogen™, California, CA, USA), in triplicate/fish.

For histological and immunohistochemical analyses, three pellets (each corresponding to a pool of cells from four wells) were obtained after centrifugation at 160 g for 5 min, and fixed with 10% neutral buffered formalin (Epredia, Breda, The Netherlands) for 24 h. Cells from four wells were collected for molecular analysis, and the obtained pellet was snap-frozen in liquid nitrogen and stored at − 80 °C.

### Hematoxylin and eosin (H&E) histological analyses

After 24 h of fixation, the gonads, liver fragment and hepatocyte pellet from each fish (n = 5 females and n = 5 males) were processed by a routine histological procedure in an automatic processor (Leica TP 1020, Leica Biosystems, Wetzlar, Germany). Before processing, the cell pellets were embedded in Histogel™ (Epredia, Breda, The Netherlands) according to the manufacturer’s instructions.

After processing, samples were embedded in paraffin, sectioned at 3 µm in a fully automated rotatory RM2255 microtome (Leica Biosystems, Wetzlar, Germany), and placed on silane-coated slides. The paraffin blocks were trimmed until reaching a large sampling area. For the liver and cell pellets, five slides were sectioned, with four sections each: one slide for H&E staining and the remaining for immunohistochemistry. As for the gonads, one slide (with two sections) was obtained for H&E staining. All sections were observed and photographed with a BX50 microscope (Olympus, Tokyo, Japan), coupled with a DP21 camera (Olympus, Tokyo, Japan).

All H&E gonad sections were staged according to an OECD criterion (OECD 2010) by differentiating five stages for males (0—undeveloped; 1—early spermatogenic; 2—mid-spermatogenic; 3—late spermatogenic and 4—spent) and six stages for females (0—undeveloped; 1—early development; 2—mid-development; 3—late development; 4—late development /hydrated and 5—post ovulatory).

### Immunohistochemistry

Immunohistochemistry analyses were performed on slides obtained from all livers and respective primary hepatocyte cultures. Slides were deparaffinized, hydrated, and washed in tap water. Then, antigen retrieval was applied distinctly depending on the antibody used (Supplementary Table S1) and according to previous optimizations (Lopes et al. 2020). The same antibodies or clones were used in other fish studies (including brown trout), which confirms their specificity (Lopes et al. 2020; Arukwe and Røe 2008; Munchrath and Hofmann 2010). To ensure comparability, immunohistochemistry for each antibody was carried out simultaneously on all slides (liver fragments and hepatocyte pellets). After antigen retrieval, endogenous peroxidase was blocked using 3% hydrogen peroxide in methanol for 10 min. Then, the slides were washed with tris-buffered saline (TBS), and the protein block reagent from the NovoLink™ Polymer Kit (Leica Biosystems, Wetzlar, Germany) was added for 5 min. After two washes in TBS, the slides were incubated with the primary antibodies diluted in PBS phosphate buffer saline (PBS) with 0.5% bovine serum albumin (BSA) (Nztech, Auckland, New Zealand) for 2 h at room temperature (20° C) in a humidified chamber. Then, the following steps were performed with the same kit according to the manufacturer’s instructions. The antibody reactions were detected by adding 3,3’-diaminobenzidine (DAB) for 2 min, and the slides were then counterstained for 1 min with Mayer’s hematoxylin, dehydrated, cleared and mounted with Q Path^®^ Coverquick 2000 (VWR Chemicals, Radnor, Pennsylvania).

For each antibody, liver sections from adult female brown trout (obtained in prior studies) were used as positive controls. Negative controls were included in each slide using the antibody dilution buffer (PBS with 5% BSA) instead of the primary antibody. The slides were observed and photographed using a BX50 microscope (Olympus, Tokyo, Japan) coupled with a DP21 camera (Olympus, Tokyo, Japan). The immunohistochemistry slides were evaluated using a semiquantitative analysis of the signal and classified into five immunostaining scores (IS): no signal (IS 0), low (IS 1), medium (IS 2), strong (IS 3), and very strong (IS 4) immunostaining.

### RNA extraction and cDNA synthesis

From each fish, the liver fragment and one hepatocyte pellet were used to extract the total RNA with an illustra™ RNAspin Mini RNA isolation Kit (GE Healthcare, Chicago, IL, USA), which includes a DNase I treatment step to avoid genomic DNA contamination of samples. RNA purity and quantification were checked in a Multiskan™ GO microplate spectrophotometer (Thermo Scientific, Vantaa, Finland), with a μDrop™ Plate and a SkanIt Microplate Reader software version 4.1. cDNA syntheses were made with an iScript™ Reverse Transcription Supermix Kit (BioRad, Hercules, CA, USA) using 300 ng of total RNA from each sample per 20 µL of total reaction volume for a total volume of 20 µL.

### Quantitative real-time polymerase chain reaction (RT-qPCR)

A CFX Connect real-time PCR detection system and CFX Manager software version 3.1 (Bio-Rad, Hercules, CA, USA) were used for RT-qPCR. Each qRT-PCR included duplicates of each sample and no template controls. The reaction mixtures (total volume of 20 µL) consisted of 5 µL of diluted (1/5) cDNA, 10 µL of iQ™ SYBR^®^Green Supermix (Bio-Rad, Hercules, CA, USA), and 200 nM of specific primers for each gene (Table 1). Melt curve analysis was carried out to determine the PCR product's purity and specificity at the final stage of the amplification cycles. Expression of the target genes was normalized by the Pfaffl method (Pfaffl 2001). The geometric mean of the two reference genes (*ribosomal protein L8*—*rpl8* and *beta-actin*—*β-actin*) was used for normalization (Table 1).

**Table 1:** Primer sequences, annealing temperatures (T), and efficiencies (E)

Gene	Primer sequence (5′–3′)	T (°C)	E (%)	References
*ERα*	F—GACATGCTCCTGGCCACTGT R—TGGCTTTGAGGCACACAAAC	61.6	91.2	Körner et al. (2008)
*ERβ*	F—TGTGGACCTGTGCCTGTTC R—ACATGAGCCCTAGCATCAGC	66.5	103.3	Körner et al. (2008)
*VtgA*	F—AACGGTGCTGAATGTCCATAG R—ATTGAGATCCTTGCTCTTGGTC	62.9	99.0	Körner et al. (2008)
*ZP2.5*	F—ATCAATAACCACAGCCACAATG R—ACCAGGGACAGCCAATATG	55.0	99.0	Uren Webster et al. (2015)
*β-actin*	F—TCTGGCATCACACCTTCTAC R—TTCTCCCTGTTGGCTTTGG	55.0	96.1	Madureira et al. (2017)
*rpl8*	F—TCAGCTGAGCTTTCTTGCCAC R—AGGACTGAGCTGTTCATTGCG	59.0	93.8	Körner et al. (2008)

*ERα*—*estrogen receptor alpha*; *ERβ1*—*estrogen receptor beta 1;* *VtgA*—*vitellogenin A;* *ZP2.5*—*zona pellucida glycoprotein 2.5;* *β-act*—*beta actin;* *rpl8*—*ribosomal protein L8*

### Statistical analysis

Statistics and graphs were performed using the PAST 4.3 software (Hammer et al. 2001) and GraphPad Prism version 8.0.1. Fish biometry, liver and primary hepatocyte gene expression were compared between males and females with one-way analysis of variance (ANOVA), followed by Tukey’s pairwise post-hoc test. ANOVA assumptions of homogeneity of variances and normality were checked using Levene’s test and Shapiro–Wilk's test, respectively. Differences were considered significant when p < 0.05. For the analysis of the immunostaining scores, non-parametric statistical tests were used. Differences among males and females were assessed using the Kruskal–Wallis test followed by Mann–Whitney pairwise comparisons with sequential Bonferroni correction. Principal component analysis (PCA) was performed in PAST using the variance–covariance matrix of the gene expression data. The first two principal components (eigenvalues greater than 1) were used for interpretation.

## Results

### Fish biometry and gonadal staging

Table 2 details fish biometric data. Sex identification revealed an equal distribution among the 10 fish, with 5 females and 5 males. Except for gonad weight and GSI, no significant differences were obtained between males and females. Females had significantly higher gonad weights than males, resulting in a 3.4% greater GSI in females compared to males.

**Table 2:** Biometric data of male and female brown

Sex	Total weight (g)	Total length (cm)	Standard length (cm)	Liver weight (g)	HSI (%)	Gonad weight (g)	GSI (%)	K
Male	34.08 ± 10.39	14.98 ± 1.38	14.02 ± 1.48	0.56 ± 0.21	1.64 ± 0.31	0.02 ± 0.01^a^	0.07 ± 0.03^a^	0.99 ± 0.09
Female	43.86 ± 24.39	16.18 ± 2.79	14.96 ± 2.81	0.65 ± 0.27	1.64 ± 0.48	0.10 ± 0.04^b^	0.24 ± 0.04^b^	0.95 ± 0.06

HSI—hepatosomatic index; GSI—gonadosomatic index; K—condition factor. Data are expressed as mean ± standard deviation (n = 5 fish/sex). Different lower-case letters represent significant differences between sexes (*p* < 0.05)

**Fig. 1:**
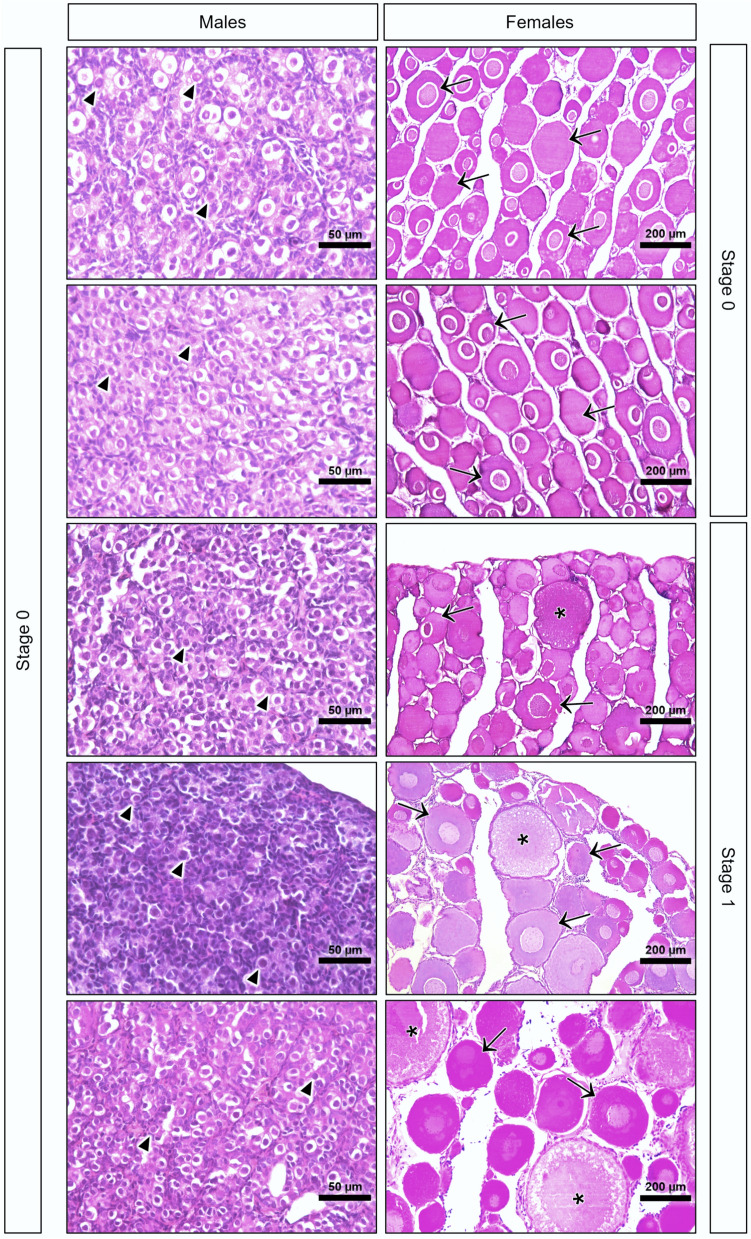
Hematoxylin & Eosin (H&E) gonad histological sections of male and female brown trouts. Black arrowheads: spermatogonia; black arrows: perinucleolar oocytes sectioned at different levels; asterisks: cortical alveolar oocytes

All males were categorized as stage 0—undeveloped since the immature stages, mainly spermatogonia, were predominated without spermatids or spermatozoa (Fig. 1). Regarding the female gonads, three females were assigned to stage 1—early development, where most of the gametes were perinucleolar oocytes, and a few were cortical alveolar ones. The other two females were classed as stage 0—undeveloped, with immature phases, and mostly displayed only perinucleolar oocytes with no cortical alveolar oocytes (Fig. 1).

### Hepatocyte viability

The hepatocyte viability after the isolation procedure was 90.5 ± 4.1% (mean ± standard deviation). After 96 h in culture, cells remained viable in all cases, with a mean viability value of 83.4 ± 7.9%.

### Liver and primary hepatocytes histological analyses

**Fig. 2:**
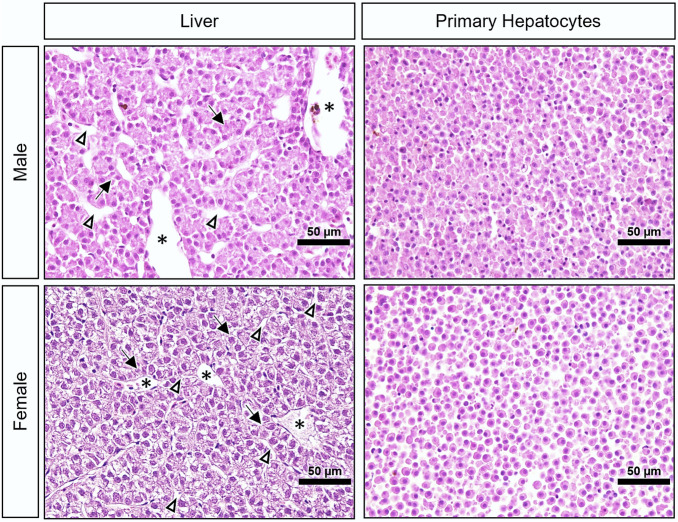
Representative images of liver and primary hepatocytes (pelleted after 96 h in culture) histological sections stained with H&E of male and female brown trout. Black arrows: hepatocyte muralium; white arrowheads: hepatic sinusoid; asterisks: venous blood vessel

Figure 2 shows representative images of the liver and primary hepatocytes stained with H&E for general morphological assessments. Hepatocytes showed regular staining and good cell preservation; neither the liver nor the cell pellets had signs of atypical cell morphology. Further, there were no observable sex-based differences.

### Immunohistochemistry

#### Positive controls

**Fig. 3:**
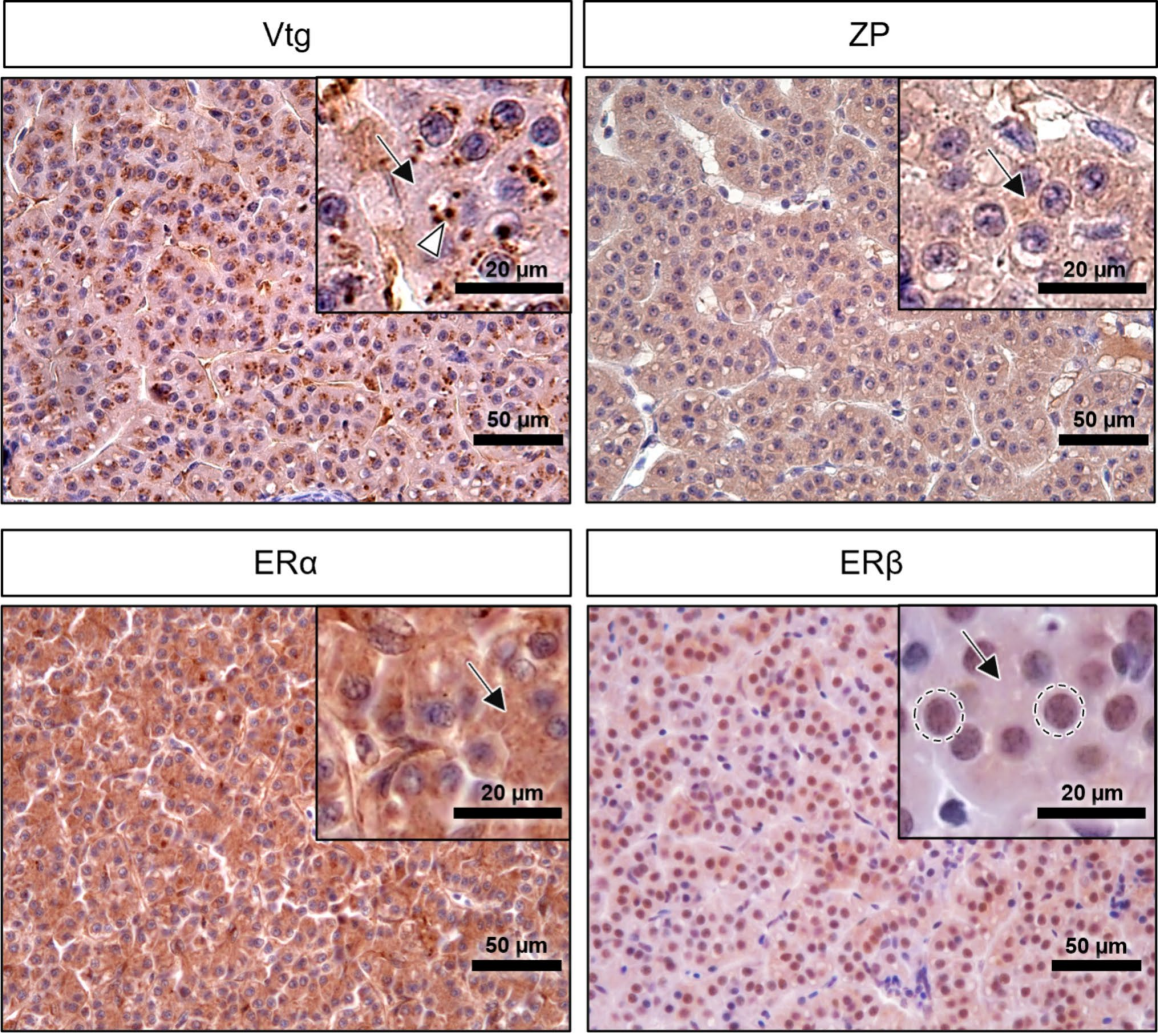
Immunohistochemistry staining in the positive controls corresponding to an adult female brown trout liver for vitellogenin (Vtg), zona pellucida glycoprotein (ZP), estrogen receptor alpha (ERα) and estrogen receptor beta (ERβ). Positive immunolabeling corresponds to a brown color signal. White arrowheads: granular cytoplasmic positive staining; arrows: diffuse cytoplasmic positive staining; and circles: nuclear immunostaining

The immunohistochemistry of positive controls for Vtg, ZP, ERα, and ERβ is illustrated in Fig. 3. Vtg was expressed in the hepatocytes of female adult brown trout livers (positive control), exhibiting predominantly strong (IS 3) to very strong (IS 4) immunostaining with a diffuse and granular pattern in the cytoplasm. Hepatocyte nuclei and cytoplasm displayed diffuse strong (IS 3) staining for ERβ, whereas ZP and ERα immunolabeling were only cytoplasmic with diffuse strong (IS 3) to very strong (IS 4) signals (Fig. 3). These outcomes contrasted with those of the negative controls carried out on the same samples; in these cases, there was either no immunolabeling or only a slight unspecific background that did not impact the immunohistochemistry analyses (data not shown).

#### Vitellogenin (Vtg)

**Fig. 4:**
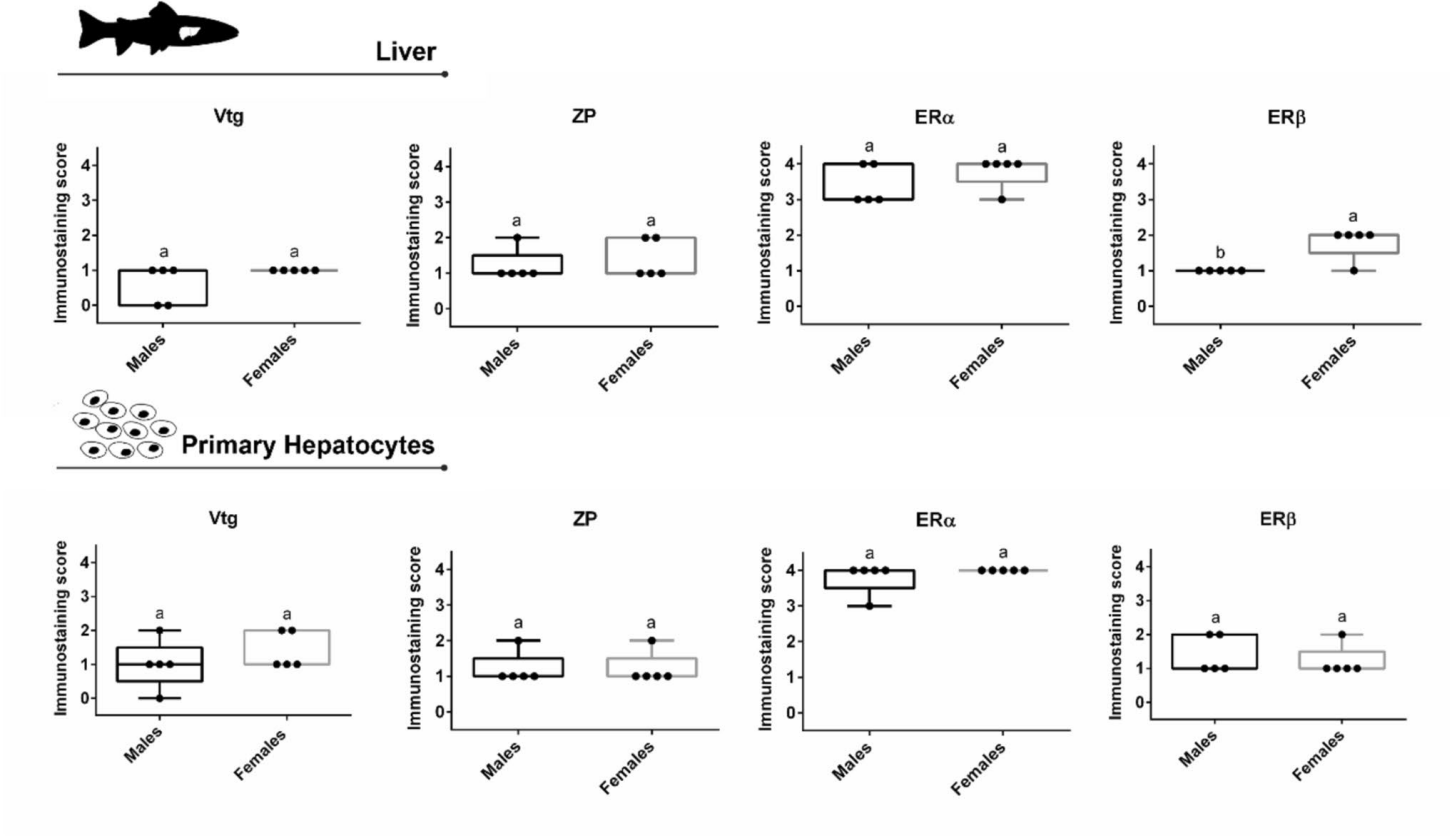
Immunostaining scores (IS) for Vtg, ZP, ERα and ERβ of liver and monolayer primary hepatocyte cultures (after 96 h in culture) obtained from brown trout (5 males and 5 females). Data correspond to the median, minimum, maximum, 25th, and 75th percentiles. Common lower-case letters indicate no significant differences between sexes (*p* > 0.05), and different letters indicate significant differences (*p* < 0.05). Vtg—vitellogenin; ZP—zona pellucida glycoproteins; ERα—estrogen receptor alpha; and ERβ—estrogen receptor beta

Overall, the Vtg immunostaining was very low (IS 0 to 1) in the liver and primary hepatocytes (Fig. 4).

**Fig. 5:**
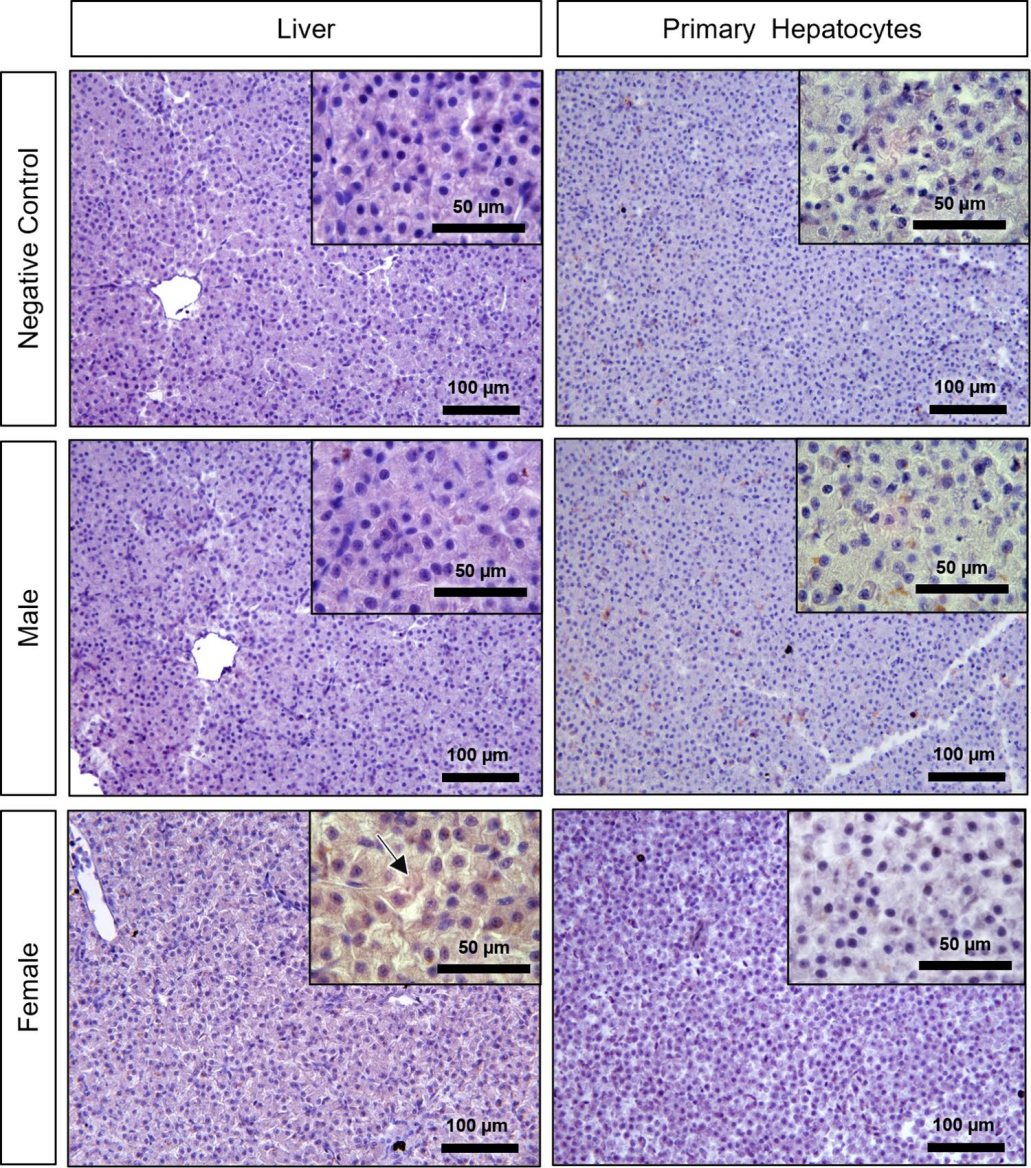
Vitellogenin (Vtg) immunohistochemistry in the negative control, male and female brown trout liver and respective primary hepatocytes (pelleted after 96 h in culture). Positive immunolabelling corresponds to a brown-colored signal. Arrow: diffuse cytoplasmic immunostaining

In most cases, regardless of sex, the pattern was similar to that found in the negative controls. Despite the resemblance, some females presented a medium (IS 2) labelling (Figs. 4, 5).

#### Zona pellucida glycoprotein (ZP)

**Fig. 6:**
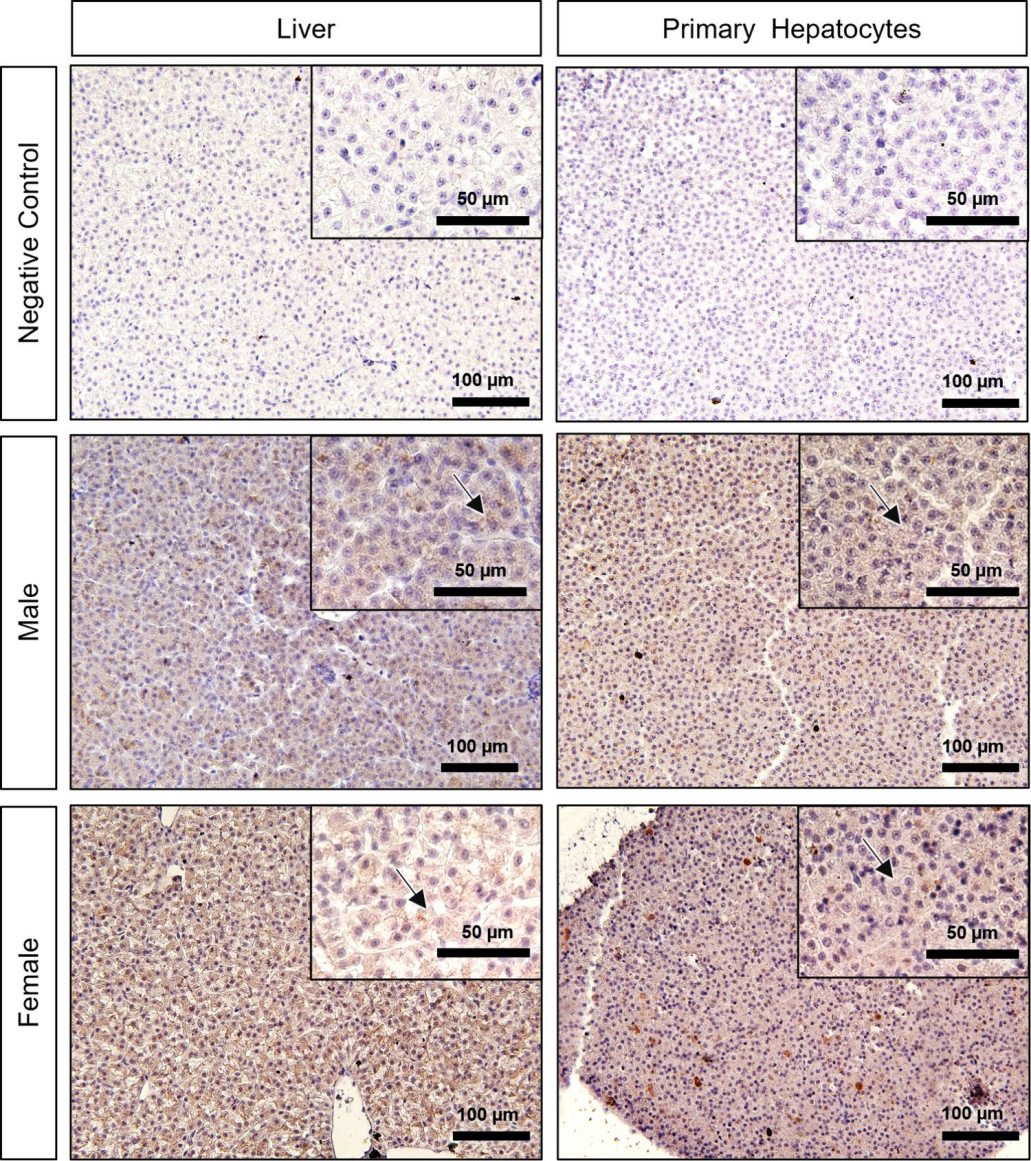
Zona pellucida glycoprotein (ZP) immunohistochemistry in negative control, male and female brown trout liver and respective primary hepatocytes (pelleted after 96 h in culture). Positive immunolabelling corresponds to a brown-colored signal. Arrows: diffuse cytoplasmic immunostaining

In both male and female samples (liver and primary hepatocytes), a low (IS 1) to medium (IS 2) level of diffuse cytoplasmic staining was observed, with negative controls serving as reference (no signal) (Figs. 4, 6). Overall, the immunoreactivity was almost equivalent in females, both in liver and primary hepatocytes, compared to male samples (Figs. 4, 6). Despite this, interindividual variability in both sexes was observed.

#### Estrogen receptor alpha (ERα)

**Fig. 7:**
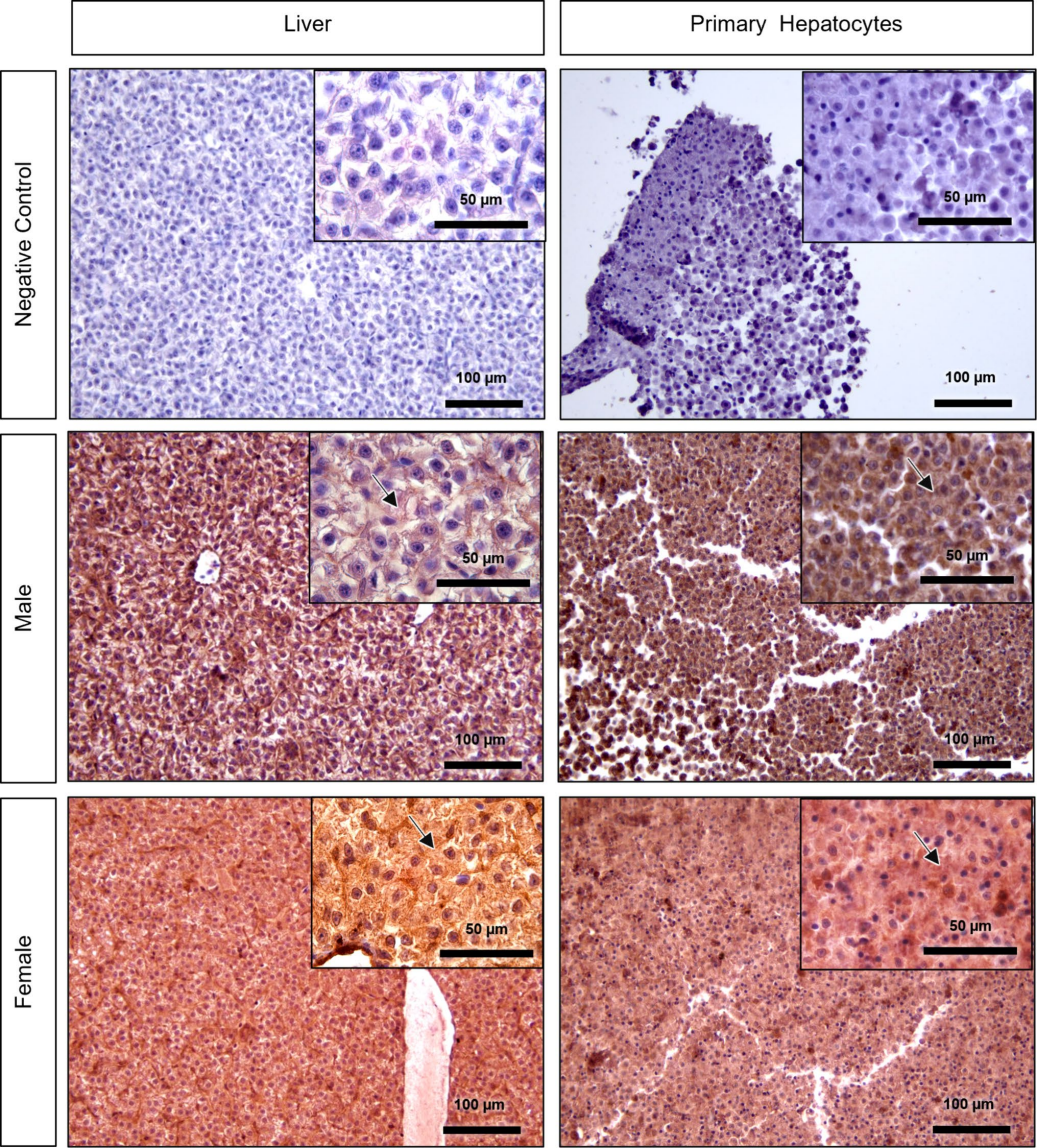
Estrogen receptor alpha (ERα) immunohistochemistry in negative control, male and female brown trout liver and respective primary hepatocytes (pelleted after 96 h in culture). Positive immunolabelling corresponds to a brown-colored signal. Arrows: diffuse cytoplasmic immunostaining

Liver and primary hepatocytes evidenced a strong (IS 3) to very strong (IS 4) diffuse cytoplasmic positive immunolabelling pattern compared to the negative control staining (Figs. 4, 7). Overall, immunolabeling intensity was higher in female samples (liver and primary hepatocytes) than in males; however, males also showed strong (IS 3) to very strong (IS 4) immunostaining. The immunostaining in the pellets tended to be stronger than in the respective liver samples (Fig. 7).

#### Estrogen receptor beta (ERβ)

**Fig. 8:**
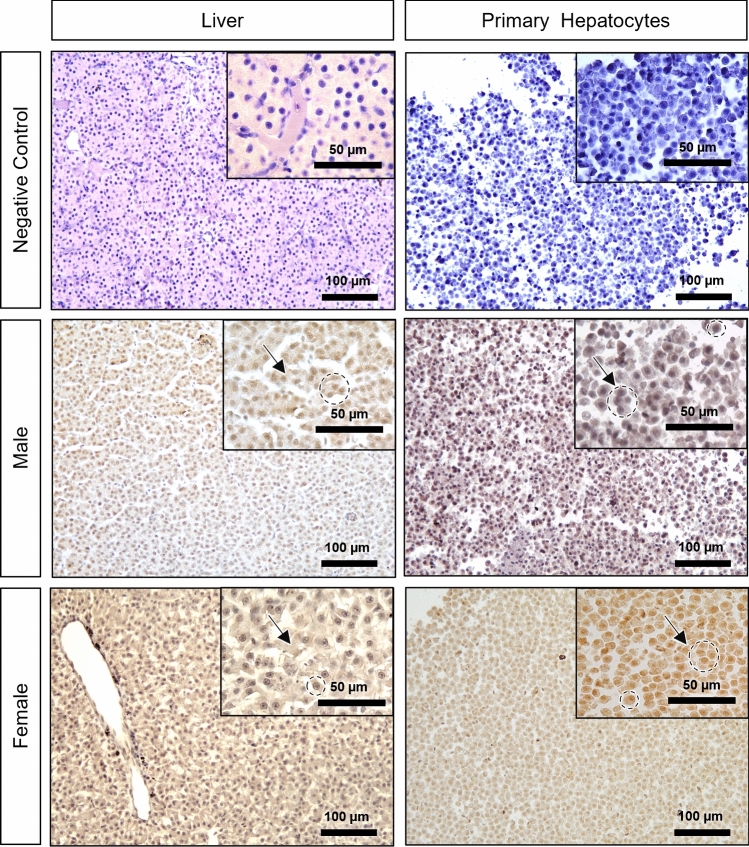
Estrogen receptor beta (ERβ) immunohistochemistry in negative control, male and female brown trout liver and respective primary hepatocytes (pelleted after 96 h in culture). Positive immunolabelling corresponds to a brown-colored signal. Arrows: diffuse cytoplasmic and circles: nuclear immunostaining

A diffuse cytoplasmic and nuclear pattern was observed in both male and female liver samples. Overall, the intensity of the immunostaining varied from low (IS 1) to medium (IS 2) signal (Fig. 4). Despite the high variability among animals, the ERβ IS in the liver was significantly higher in females than in males. Figure 8 shows examples of ERβ immunohistochemistry.

### Relative mRNA expression levels

**Fig. 9:**
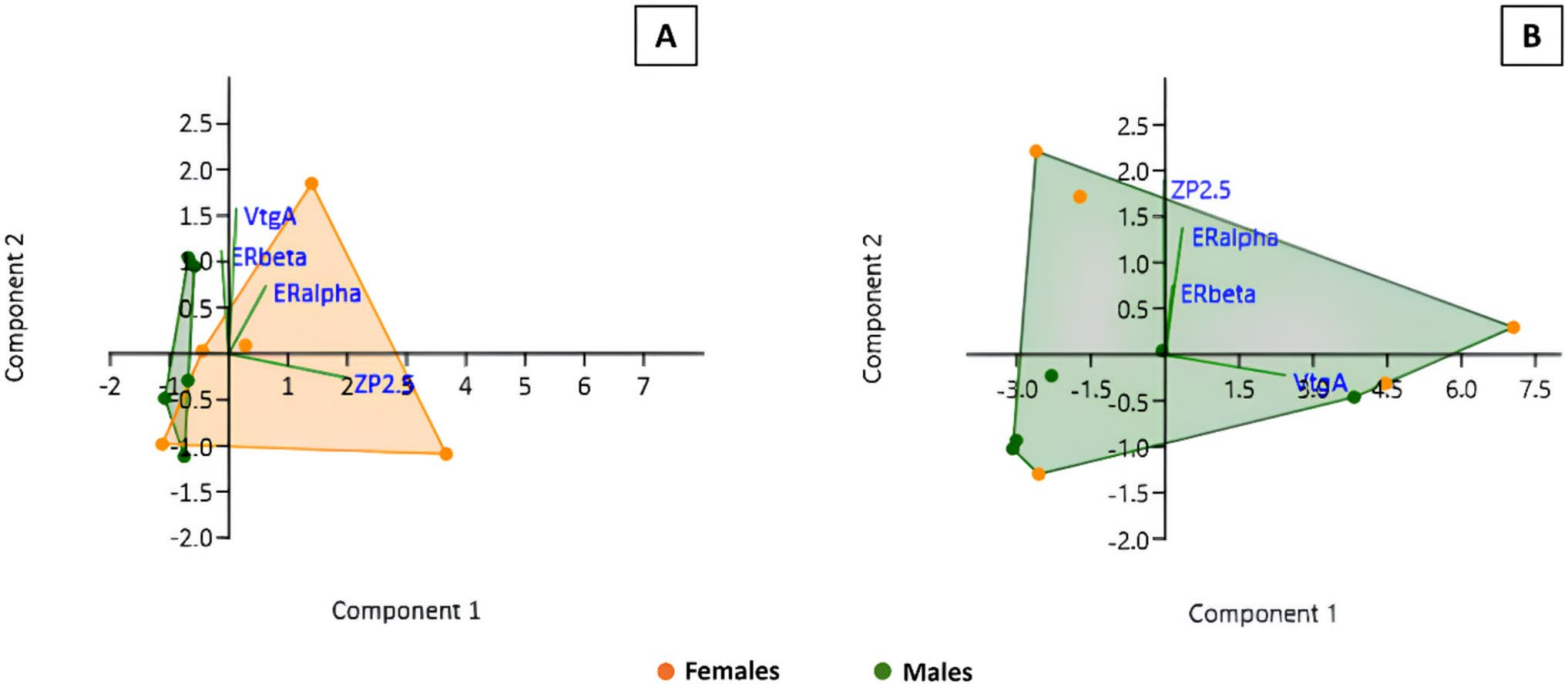
Principal component analysis scatter plots of *VtgA*, *ZP2.5*, *ERα* and *ERβ* mRNA levels of: **A** primary hepatocytes (after 96 h in culture) and **B** liver samples obtained from male (n = 5) and female (n = 5) brown trout. *VtgA*—*vitellogenin A*; *ZP2.5*—*zona pellucida glycoprotein 2.5*; *ERα*—*estrogen receptor alpha*; and *ERβ*—*estrogen receptor beta*

The PCA plot of the mRNA levels (*VtgA*, *ZP2.5*, *ERα* and *ERβ*) in primary hepatocytes from male and female donors showed two sex clusters with partial overlap (Fig. 9A). The total data variation of 93.38% is explained by PC1 (64.07%) and PC2 (29.31%). Loadings evidenced that PC1 is positively influenced by *ZP2.5* and *ERα* mRNA levels, while *VtgA* and *ERβ* had the highest positive loadings in PC2. The PCA plot of mRNA levels in liver samples showed extensive overlap between sex-specific clusters (Fig. 9B). Most of the data variation was explained by PC1 (87.12%), which was strongly influenced by *VtgA*.

**Fig. 10:**
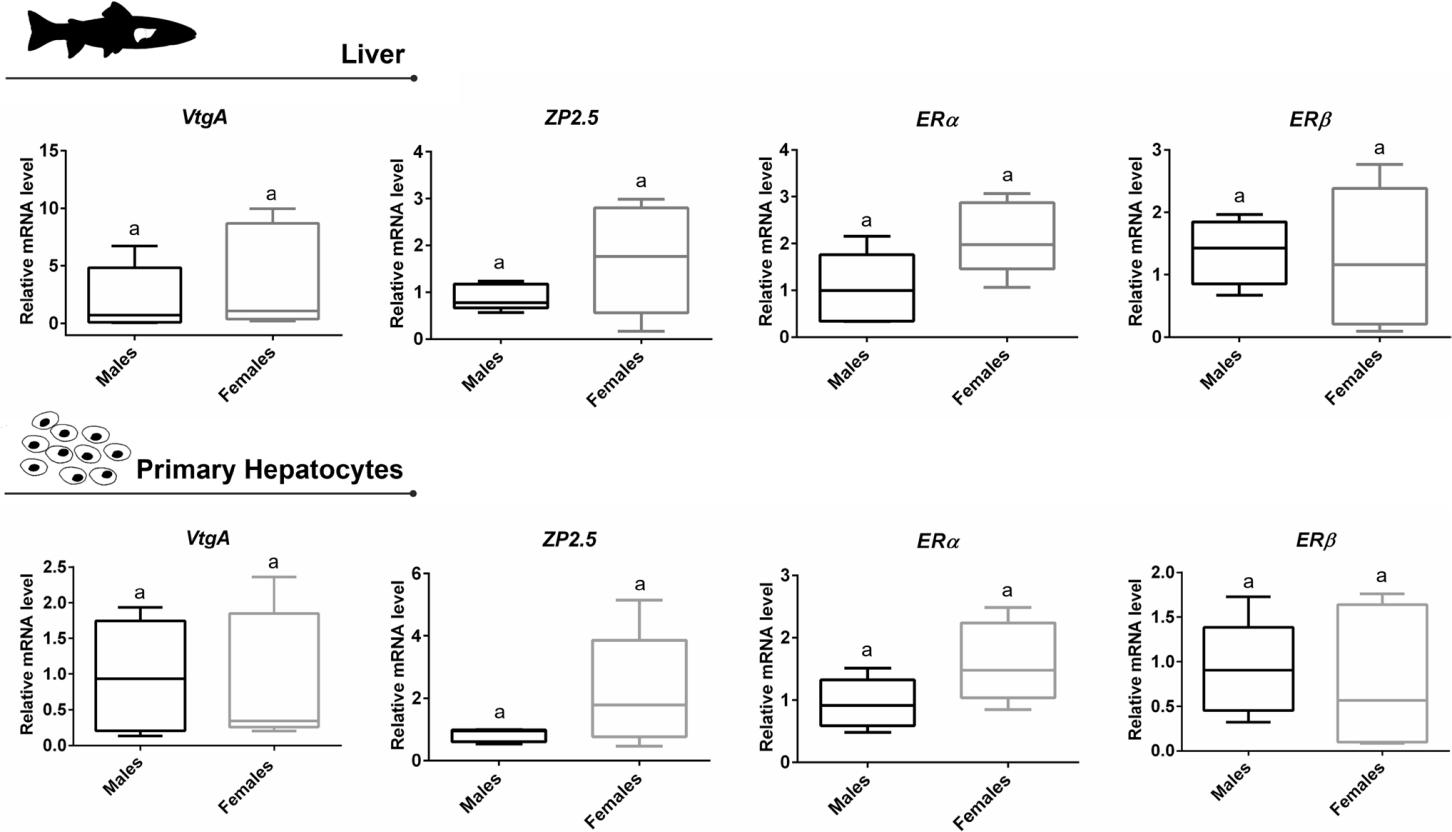
Relative mRNA levels of *VtgA*, *ZP2.5*, *ERα* and *ERβ* of brown trout liver and in monolayer primary hepatocyte cultures (after 96 h in culture) obtained from brown trout (5 males and 5 females). Data correspond to the median, minimum, maximum, 25th, and 75th percentiles. Common lower-case letters indicate no significant differences (*p* > 0.05) between sexes. *VtgA*—*vitellogenin A*; *ZP2.5*—*zona pellucida glycoprotein 2.5*; *ERα*—*estrogen receptor alpha*; and *ERβ*—*estrogen receptor beta*

In line with the partial overlap in the PCA plot (Fig. 9A), for the four estrogenic target genes (*VtgA*, *ZP2.5*, *ERα*, and *ERβ*), the relative mean mRNA levels showed no statistically significant differences between males and females, neither in the liver nor in primary hepatocytes (Fig. 10). Female livers and primary hepatocytes showed highly variable expressions, particularly in the case of *ZP2.5*, compared to males. The mRNA level patterns for all targets in primary hepatocytes closely resemble those found in the liver.

## Discussion

Primary hepatocytes are expected to show similarities to in vivo hepatic phenotypic profiles (Olsavsky et al. 2007), although there is concern over their acquisition complexity, maintenance, and the imminence of dedifferentiation over the culture time (Kiamehr et al. 2019). Moreover, in fish cell cultures, donor-related cellular variability is a known factor that has not received much attention, and it is essential, especially when studying endocrine disruption. Based on this premise, this study sought to investigate the baseline expression of estrogenic-related targets in the liver and primary hepatocytes. Four estrogenic targets (Vtg, ZP, ERα and ERβ), were analyzed in terms of gene and protein expression. It is well known that under physiological conditions, both Vtg and ZP are proteins synthesized in the fish liver in response to endogenous estrogenic stimuli (Wallace 1985; Oppen-Berntsen et al. 1992). Moreover, previous studies using primary brown trout hepatocytes showed increased *VtgA* and *ERα* gene expression after exposure to 17α-ethinylestradiol, confirming that the model is estrogen-responsive (e.g., Madureira et al. 2015; Lopes et al. 2020).

Every fish in this study had its biometric data and gonad developmental stage described. All the fish belonged to the same batch. However, it was found that gonadal maturation status varied among females, with over half in an early developmental stage, and that all the males were categorized as undeveloped. Despite these differences, significant hormone-regulated hepatic alterations were not expected in this juvenile cohort, since the known liver seasonal plasticity mainly occurs in adult brown trout breeders during vitellogenesis (Rocha et al. 2010).

Immunohistochemistry showed distinct labelling for each protein target, resembling those previously documented for the same species (Lopes et al. 2020). Generally, a higher intensity of immunostaining was observed for ZP compared to Vtg (irrespective of sex), consistent with findings from other studies indicating that the regulatory mechanisms and responsiveness to estrogens are distinct (Arukwe et al. 1997; Berg et al. 2004). Immunostaining for ERα was notably strong to very strong, and it was the only target where females exhibited a slightly more intense staining than males (concerning the liver and primary hepatocytes).

For the relative mRNA levels of *VtgA*, *ZP2.5*, *ERα* and *ERβ*, we did not detect significant differences across donor samples, both for the liver and primary hepatocytes. Yet, we found that the mean relative mRNA levels of *ZP2.5* and *ERα* were approximately 1.9 and 1.6-fold and 2.7 and 1.7-fold greater in females’ liver and primary hepatocytes than in males, respectively. *Vtg* induction, *ERα* up-regulation, and *ERβ* activation were correlated in a prior study using goldfish primary hepatocytes, suggesting that Vtg regulation depends on both ERs, with the amount of each ER varying according to the breeding season (Nelson and Habibi 2010). In the present study, *VtgA* and *ERβ* mRNA levels overlapped entirely in primary hepatocytes across all individuals, which is consistent with evidence that Vtg synthesis is mainly mediated by ERβ, as described in rainbow trout (Leaños-Castañeda and Kraak 2007).

Although interindividual variability in basal gene and protein expression was expected in juvenile fish, given natural differences in development and endocrine status among specimens, the absence of significant sex-based differences suggests that primary brown trout hepatocytes may represent a robust and reproducible baseline model for estrogen testing. The lack of statistically supported sex differences likely reflects the immature endocrine status of the juveniles used, during which estrogenic pathways are not yet fully activated or sexually differentiated. Under these conditions, normal biological heterogeneity does not appear to compromise experimental reproducibility. Altogether, these outcomes emphasize the need to document and account for interindividual variability when interpreting baseline estrogenic signatures in primary hepatocyte cultures, while also highlighting that this juvenile hepatocyte model is a robust and reliable baseline for assessing estrogenic compounds.

The subtle (non-significant) results for the targets described may be related primarily to two factors. First, basal levels in juveniles would not be expected to be elevated since the gonadal stage for both sexes demonstrated immature phases, so circulating sex-hormone levels would be negligible. Second, individuals with developing ovaries exhibited a broader range of gonad stages (from stage 0 to stage 1), which should reflect the start of progression towards adulthood and could partially, and logically, explain the greater variability in the gene expression levels, and our "inability" to prove differences attributed to reproductive status at this juvenile stage. Previously, Bickley et al. (2009) also reported a higher estrogen response variation in the case of hepatocytes isolated from female common carps, and they were not able to prove significant sex-based differences (Bickley et al. 2009). Also, according to Bjerregaard et al. (2008), brown trout's age and size had an impact on their plasma Vtg levels (Bjerregaard et al. 2008). In the same rationale, fish biometry and gonad staging—which are rarely included in routine experimental descriptions—should be carefully considered when selecting donors for studies using primary hepatocytes.

Some experiments used juvenile or immature fish to obtain primary hepatocyte cultures to mitigate or supposedly abolish the potential influence of reproductive hormones on cell cultures, e.g., Hultman et al. (2015a), Winzer et al. (2002a), Lopes et al. (2020). Nonetheless, if fish were not fully detailed regarding the gonad stage, as has been done here, those concepts might not be accurately defined.

In this study, hepatocytes were cultured for 96 h, as toxicity assays often benefit from more extended exposure periods that more closely resemble in vivo conditions. Further, that period has been successfully optimized for brown trout primary hepatocyte cultures (Madureira et al. 2015; Lopes et al. 2020). However, despite evidence that Vtg mRNA and protein expression after estrogenic stimuli may increase before 24 h, consistent induction was only noted at 48 h to 96 h, although extending the exposure to 96 h typically involves a new estrogenic input via culture medium exchange (Hultman et al. 2015b). In the context of the present study, this information could signal that our results were obtained on a stable plateau of expression levels, and therefore, it could be argued that it would be unfeasible to infer reproductive status-related differences in basal levels. As a counterargument, the response pattern observed across all targets in the liver (sampled before isolation) and primary hepatocytes (sampled after 96 h in culture) was similar, undermining the hypothesis that sampling time is masking the expression levels obtained in this study.

This study explicitly accounted for basal interindividual biological variability, using a sex-balanced design based on gonadal development, which enabled an investigation of possible differences in estrogenic markers—an aspect often overlooked in fish toxicology studies (e.g., Zablocki da Luz et al. 2024; Zhou et al. 2006). In addition to not compromising the main inferences, the number of animals was chosen here to minimise their use following ethical requirements and in line with the sample sizes commonly used in toxicological studies involving primary fish hepatocytes (e.g., Winzer et al. 2002b; Hultman et al. 2015b; Maradonna et al. 2013). Although no sex-associated different patterns were statistically demonstrated under basal conditions, this does not exclude the eventual existence of sex-specific responses of primary brown trout hepatocytes under estrogenic stimulation. Dedicated exposure experiments under standardized conditions and sufficient replication are warranted to determine whether males and females differ in their hepatic responses to estrogenic compounds.

## Conclusion

Overall, our data suggest that biological differences among juvenile brown trout donors can introduce baseline variability in primary hepatocyte cultures. While we did not directly test how this variability may influence toxicological assay outcomes, our findings highlight the need to document and control donor-related factors when designing and interpreting such studies. Accordingly, it is recommended that future studies should consider: (1) if using immature fish, the gonadal maturation stage must be validated through histological examination, and its potential influence investigated; (2) in the case of mature fish, select individuals at comparable stages of gonadal development; (3) if a single sex is used, preference could be given to male donors (ideally from the same batch)—expected to have less variable gonadal development—as this may reduce baseline variability in estrogenic endpoints.

## Supplementary Information

Below is the link to the electronic supplementary material.

Supplementary file1 (DOCX 14 kb)

## Data Availability

The datasets generated during and/or analyzed during the current study are available from the corresponding author on reasonable request.
