# Supplementary material for: Baseline estrogenic activity in juvenile brown trout liver and primary hepatocytes: integrated molecular and immunohistochemical analysis
Source: J Comp Physiol B. 2026 Apr 1;196(2):177–92. doi: 10.1007/s00360-026-01657-0 (PMC13233662; doi:10.1007/s00360-026-01657-0)
Supplement: Supplementary file 1 — Supplementary file1 (DOCX 14 kb) [file 360_2026_1657_MOESM1_ESM.docx]

**Table S1**. Antibodies, dilutions and antigen retrievals used for immunohistochemistry protocols.

| **Antibody** | **Clonality** | **Dilution** | **Buffer** | **Antigen Retrieval procedure** | **Reference** |
| --- | --- | --- | --- | --- | --- |
| **Vtg**  Bertin Bioreagent  Ref: V01409201 | Polyclonal rabbit anti-arctic char vitellogenin | 1:2500 | Tris-EDTA 10 mM Tris Base, 1 mM EDTA, pH 9, 0.05% Tween 20 | The buffer was preheated in the microwave at 700W until reaching the boiling point. Then, the slides were immersed (15 min). | Arukwe and Røe 2008 |
| **ERα**  Millipore  Ref: 06-935 | Polyclonal rabbit anti-human | 1:100 |  |  | Munchrath and Hofmann 2010 |
| **ERβ**  Enzo Life Sciences  Ref: ALX-210-180 | Polyclonal rabbit anti-human | 1:1000 |  |  | Munchrath and Hofmann 2010 |
| **ZP**  Bertin Bioreagent  Ref: Z03402202 | Polyclonal anti-  purified eggshell proteins from Atlantic salmon | 1:3000 | Citrate buffer 0.01 M pH 6 | The buffer was pre-heated in the pressure cooker until reaching the boiling point. Then, the slides were immersed (3 min) after maximum pressure was reached. | Arukwe and Røe 2008 |
